# Tunable Quantum Photoinitiators for Radical Photopolymerization

**DOI:** 10.3390/polym13162694

**Published:** 2021-08-12

**Authors:** Shubhangi Shukla, Prem C. Pandey, Roger J. Narayan

**Affiliations:** 1 Joint Department of Biomedical Engineering, University of North Carolina, Raleigh, NC 27599, USA; sshukla3@ncsu.edu; 2 Department of Chemistry, Indian Institute of Technology (BHU), Varanasi 221005, India; pcpandey.apc@iitbhu.ac.in

**Keywords:** quantum photoinitiators, upconverting nanoparticles, quantum confinement effect, semiconductor nanocrystals

## Abstract

This review describes the use of nanocrystal-based photocatalysts as quantum photoinitiators, including semiconductor nanocrystals (e.g., metal oxides, metal sulfides, quantum dots), carbon dots, graphene-based nanohybrids, plasmonic nanocomposites with organic photoinitiators, and tunable upconverting nanocomposites. The optoelectronic properties, cross-linking behavior, and mechanism of action of quantum photoinitiators are considered. The challenges and prospects associated with the use of quantum photoinitiators for processes such as radical polymerization, reversible deactivation radical polymerization, and photoinduced atom transfer radical polymerization are reviewed. Due to their unique capabilities, we forsee a growing role for quantum photoinitiators over the coming years.

## 1. Introduction

The use of novel photoinitiators (PIs) for free-radical polymerization has attracted significant attention from the scientific community [1,2,3,4,5]. Quantum PIs, quantum-confined nanoscale crystals with semiconductor properties, have received interest for use in photopolymerization, due to their precisely tunable properties as a function of structural and surface engineering [1,2,3]. Current advancements in synthetic procedures have led to the development of a variety of quantum PIs with characteristic photoinitiating properties [4,5,6,7,8,9,10]. The surface moieties introduced in the course of nanocrystal synthesis facilitate control over skeletal arrangements, dispersibility, and reactivity at the molecular level [1,2,3,4,5,6,7,8,9,10]. Postsynthetic surface modifications enable the dispersal and stabilization of nanocrystals PIs in formulations for many applications [5,6,7,8,9,10,11,12,13,14,15,16,17,18,19,20].

**Figure 1:**
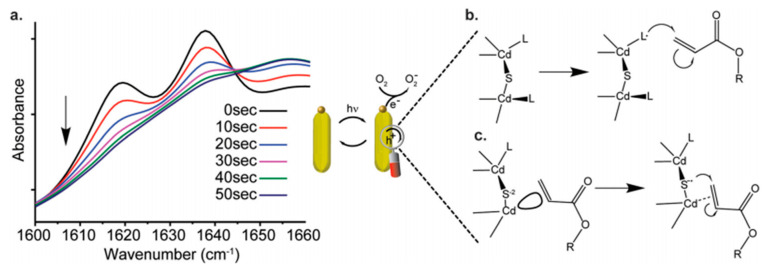
Insights into the mechanism. (**a**) FTIR spectra of the polymerization process of hydroxyethyl acrylate (HEA) using CdS-S^2−^ in the presence of water. The C=C doublet at 1619 cm^−1^ and 1637 cm^−1^ disappears with illumination time. (**b**) Proposed Mechanism 1: the initiation is carried out by hole transfer from the semiconductor to the monomers via surface coating mediation. (**c**) Proposed Mechanism 2: the double bond is coordinated by the cation, followed by a hole transfer from the anion-localized state [10].

**Figure 2:**
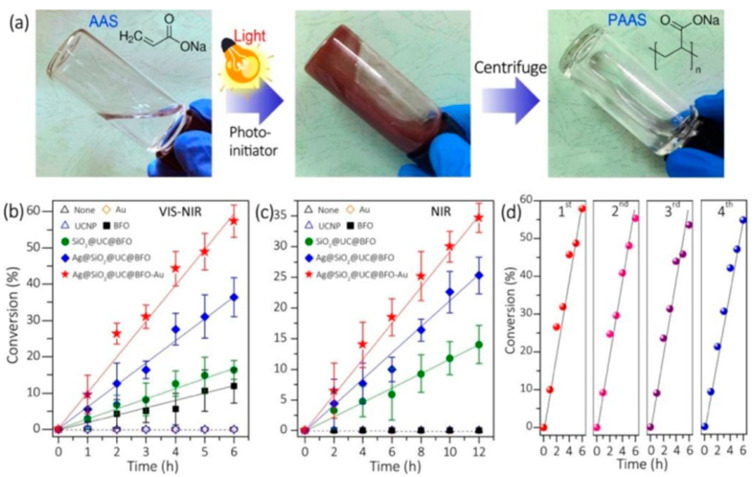
(**a**) Pictures of acrylic acid sodium (AAS) aqueous solution without NP photo-initiator (left), after polymerization reaction before (middle), and after (right) removal of Ag@SiO_2_@UC@BFO-Au initiator. Conversion of AAS using different NPs as initiators under white light (**b**) and near infrared (NIR) light (**c**) (the lines are to guide the eyes). (**d**) Repeated runs for the polymerization using Ag@SiO_2_@UC@BFO-Au under VIS light [31].

Several classes of quantum PIs have been developed and evaluated for polymerization, with various absorption and excitation wavelength windows [15,16,17,18,19,20]. The mode of action of most of the quantum PIs is generally similar to type I radical initiators, with highly conjugated aromatic moieties; others mimic the activity of type II initiators [5,6,7,8,9,10]. Loir et al. indicated that two pathways exist for initiation in quantum PIs, which involve surface mediated hole transfer (Figure 1) [10]. Typical quantum PIs can be classified as semiconductor nanoparticles (NPs), such as TiO_2_, ZnO, and CdS NPs [3,4,5,6,7,8,9,10,11,12,13,14,15,16]; hybrid photoinitiators, including composites of metal nanoparticles (MNPs)/silanized metal (organic PIs, MNPs), fluorescent dyes, oligomeric silsesquioxane, and parent PIs, [17] semiconductor NPs (metal/graphene oxide) [18,19,20,21,22,23], and organometallic nanoparticles; panchromatic photoinitiators, namely upconverting nanoparticles (UCNPs) [24,25,26,27,28,29,30,31,32,33,34] and plasmonic nanoparticle composites (e.g., Ag@SiO_2_@UC@BFO–Au core@triple-shell); near-infrared photoinitiators, such as luminescent lanthanides (e.g., Ln^3+^, Yb^3+^, Er^3+^, and Ho^3+^) and doped nanomaterials in a crystalline host lattice (NaYF_4_); [31] magnetic nanoparticles, such as Fe_2_O_3_; and metal core-shell nanoparticles (e.g., Ag@AgCl nanocubes) [35,36]. Figure 2 describes the polymerization of acrylic acid sodium (AAS) using the Ag@SiO_2_@UC@BFO-Au photoinitiator.

In general, nanoscale PIs are efficient for photo-curable polymerization applications in aqueous solutions and ultraviolet light (UV)-dependent stereolithography 3D printing [17,25]. They have superior conversion rates, a low risk of migration, exceptional cross-linking behavior, and good compatibility with aqueous media, owing to a large absorbance cross-section in UV–Vis region [20,21,22,23,24,25,26,27,28,29,30,31,32,33,34,35,36,37,38,39,40,41]. Quantum PIs enable the migration issue to be minimized due to their higher molecular weights. For example, nanocrystal PI-assisted photopolymerization of methyl methacrylate (MMA) was demonstrated on a zinc oxide surface [1]. Photon excited electron-hole pair generation in semiconductor PIs with a slow recombination step and homogeneity in the system enables rapid polymer formation. The charge carriers effectively control the concentration of oxidative species generated and also retard the annihilating function of the oxygen species. Quantum PIs are also used for two-photon (2P) absorption-based printing approaches [42,43,44,45,46,47,48,49,50,51,52,53]. In addition, 2P polymerization processes involve the use of femtosecond laser radiation at a wavelength below the bandgap to excite the quantum PI. Quantum PIs have recently been used to study novel polymerization techniques, such as atom transfer radical polymerization (ATRP), reversible deactivation radical polymerization (RDRP), and reversible addition–fragmentation chain transfer (RAFT) [33,54]. This review considers different approaches used to develop quantum PIs for photopolymerization applications. In particular, the optoelectronic properties, cross-linking, properties, and functionality of these photoinitiators are described.

## 2. Categories of Quantum Photoinitiators

### 2.1. Semiconductor QPIs

Quantum photoinitiators based on semiconductor nanocrystals are considered a viable alternative to traditional organic photoinitiators with low molecular weights. Semiconductor nanocrystals have attracted attention due to their capacity to function as photocatalysts for many types of chemical reactions; these materials offer unique advantages, such as efficient light-harvesting activity, tunable properties, and large surface area-to-volume ratios [2]. These nanocrystals exhibit quantum confinement effects; the properties of these materials may be modified by synthetic control over nanocrystal size, shape, and composition [2].

Semiconductor quantum dots (QD) are solution-dispersible nanocrystals, which have found use as photocatalysts for light-induced polymerization. QDs exhibit strong absorption in the UV-visible range, with large extinction coefficients (ε > 10^5^ M^−1^ cm^−1^) [14], large specific surface area values that allow for the interaction with multiple substrates, and higher photostability than organic-based photocatalysts and transition metal complexes [14], Semiconductor suspensions were first used for photopolymerization by Kuriacose et al.; ZnO powders were used for photopolymerization of methyl methacrylate (MMA) in water [1]. The effect of the oxygen level on polymerization was investigated; higher amounts of oxygen were associated with a lower molecular weight and a larger number of chains. On the other hand, too much oxygen in the solution suppressed the propagation step; this phenomenon was reported to be a common limitation for polymerization initiated by oxidative anionic species-releasing photoinitiators. In addition, bulk size initiators were found to be less effective for polymerization. Semiconductor nanocrystals of TiO_2_, ZnO, and CdS were found to be better candidates for initiation, due to the fact that they are associated with less light scattering and higher surface area to volume ratios [2]. Among these three nanocrystals, CdS was observed to provide the maximum yield of polymerization, as the conduction band energy in CdSs is at the highest level; the excitation of CdS QDs possibly occurs in visible region. In contrast, ZnO QDs are excited by UV illumination at 367 nm and higher energies; photoinitiation is energy intensive for ZnO nanocrystals, due to their wide bulk band gap. Additional studies have been conducted on facilitating absorption by ZnO in visible region through hybrid photoinitiators that combine ZnO QDs with dyes, fluorescent pigments, and organic photoinitiators [2]. A number of reports are available on the use of semiconductors as radical initiators, and which describe their unique advantages, such as a broad and tunable excitation window and limited migration.

For example, Pinkas et al. studied the effect of the morphology of zinc oxide (ZnO) nanocrystals on polymerization efficiency [3]. Experiments were performed using aqueous as well as solvent-free formulations, to compare the effect of two distinct shapes of particles (e.g., rod- and pyramidal-shapes) with similar particle volumes and surface area values. ZnO rods showed a clear advantage over the pyramids for use as PIs; the enhanced photocatalytic activity of these materials was associated with higher rates of oxygen consumption and formation of reactive species [3]. The efficiency of nanorods was confirmed in terms of their photocatalytic activity for dye reduction and oxidation, oxygen consumption, and reactive oxygen species formation. 

Pawar et al. described the use of a hot-injection procedure, followed by selective metal growth for processing cadmium sulfide (CdS) nanorods with gold tips [44]. The materials were dispersed in distilled water in the presence of polyethylenimine (PEI), which amplified their photocatalytic activity and facilitated reactive oxygen species (ROS) formation. The as-synthesized nanocrystals exhibited matchstick shapes; the CdS nanorods possessed single gold tips [44].

CdS–Au semiconductor–metal hybrid nanoparticles were used as photoinitiators for digital light processing (DLP) 3D printing with 385–405 nm light. The polymerization kinetics was compared with those of simple CdS nanorods and a commercially water-soluble PI, Irgacure 2959. These nanoparticles were both noted to consume inhibitory dissolved oxygen and create hydroxyl radicals during polymerization.

**Figure 3:**
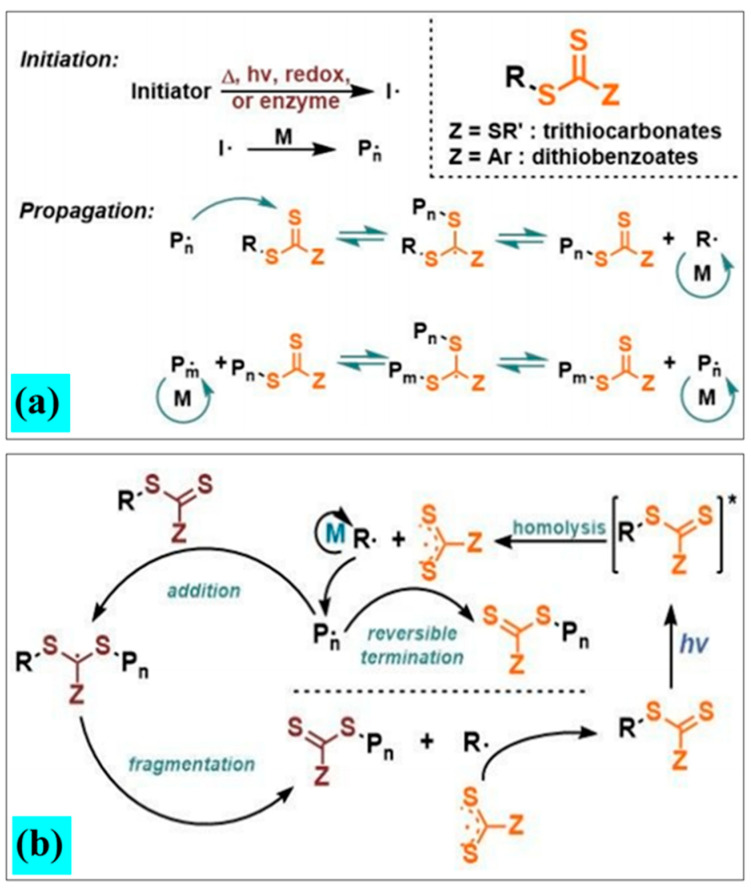
(**a**) The general mechanism for “living” radical polymerization, involving thiocarbonylthio compounds. (**b**) The general mechanisms for photoinduced RAFT polymerization, in which RAFT agents are initiators and CTAs simultaneously [39].

Zhu et al. evaluated the use of cesium-doped lead halide perovskite (CsPbBr_3_) nanocrystals as band-edge-tunable photocatalysts, to understand photoinduced electron/energy transfer–reversible addition–fragmentation chain transfer (PET–RAFT) polymerization [54]. The chain end fidelity of the obtained polymers was analyzed by proton nuclear magnetic resonance (1 H NMR) and UV–Vis spectroscopy [54]. The photocatalytic function of CsPbBr_3_ NCs for PET–RAFT polymerization was demonstrated; 2-(n-butyltrithiocarbonate) propionic acid (BTPA) served as the chain transfer agent (CTA) and methyl acrylate (MA) served as monomer [54]. The polymerization was performed using 1 wt% photocatalyst loading, with respect to the amount of monomer under blue LED irradiation (power = 10 mW/cm^2^); toluene was used as the solvent. Activation of the BTPA chain end was associated with effective electron transfer between the BTPA and the perovskite NCs. The setup resulted in polymers that exhibited a narrow dispersity (Đ = 1.02–1.13) and a high degree of chain-end fidelity (Figure 3). Furthermore, the large two-photon absorption cross-section of CsPbBr_3_ promoted straightforward activation in the presence of near-infrared laser pulses [54].

Cheng et al. studied the use of TiO_2_ as a photoredox catalyst for the PET–RAFT polymerization of methyl methacrylate (MMA). Since TiO_2_ has strong absorption between 300 nm and 400 nm, TiO_2_ generates electrons (e^−^) and holes (h+) upon UV irradiation. The valence-band hole (h+) serves as an oxidant and the The conduction-band electron serves as a reducer [55]. TiO_2_ was shown to be able to reduce 4-Cyanopentanoic Acid Dithio benzoate (CPADB) via a photoinduced electron transfer (PET); the resulting free radicals initiate the polymerization of MMA via the RAFT process [55]. The ultraviolet irradiation-based photolysis of dithioester ends had no control over the PET-RAFT polymerization process after a time of 500 min. The TiO_2_ based system controlled the molecular weights and polydispersities (PDI) of the synthesized polymer via an “ON” and “OFF” switch phenomenon [55,56,57]. They explored the effect of temporal control over the polymerization process by exposing a mixture of CPADB, MMA, and TiO_2_ to light in an alternating “ON” and “OFF” manner. No polymerization was observed in this system in the absence of light; this phenomenon indicated that the intermediate radical fragmentation and induced chain propagation were slowed or ceased in the absence of light. In contrast, the reaction continued in the presence of light; this phenomenon was associated with the fragmentation of intermediate radicals [55]. Processing of poly(methyl methacrylate)-*b*-poly(dimethylaminoethyl methacrylate) (PMMA-*b*-PDMAEMA) copolymer was demonstrated using this approach [55]. 

Hakobyan et al. demonstrated a reversible-deactivation radical polymerization process involving photo-induced electron transfer; solid Bi_2_O_3_, which is nontoxic and inexpensive, served as a photocatalyst [56]. Polymerization of N-vinylpyrrilidone (NVP) and N,N-dimethylacrylamide (DMA) was demonstrated using this approach; the polymerization process was conducted at room temperature with visible light. Both monomers underwent controlled polymerization in the presence of a xanthate, which served as a chain-transfer agent [56]. They showed that this approach, which involved an interchange of xanthate (MADIX) and a reversible addition-fragmentation chain-transfer (RAFT) polymerization approach, enabled a polymer with a narrow molecular weight distribution to be obtained. The Bi_2_O_3_ catalyst system was noted to be of low toxicity, economical, reusable, and straightforward to remove from the reaction mixture.

McClelland et al. described the use of CdSe quantum dots (QDs) as an activating agent for the PET–RAFT polymerization of acrylamides and acrylates [38]. These QDs showed high colloidal solubility and photostability, which enabled ultralow catalyst loading (<0.5 ppm) and a high degree of efficiency in terms of polymerization (>90% conversion in 2.5 h). The photocatalyst was separated from the polymer and monomer using protein concentrators; moreover, the isolated QDs that were treated for use with the subsequent processing batch did not show a significant decrease in efficiency [38].

### 2.2. Carbon-Based QPIs

Various efforts have been made to obtain carbon dots (CDs) from naturally occurring materials [58,59,60]. Owing to their straightforward preparation, capability for preparation from sustainable raw materials, and ultrastable photoluminescence, photoluminescent CDs have been utilized in bioimaging, optoelectronics, photocatalysis, and photopolymerization [58].

For example, Kiskan et al. used mesoporous graphitic carbon nitride (mpg-C_3_N_4_) alongside tertiary amine co-initiators for visible-light-induced free radical polymerization via a hybrid type II initiator approach [58]. This approach involved generating radical initiators by scavenging holes using amines and hydrogen abstraction. The surface area of the carbon nitride powder and the type of amines were shown to affect the photoinitiation efficiency associated with this approach (e.g., more basic amines enhance the efficiency of the approach). 

Similarly, Fu et al. studied RAFT polymerization using graphitic carbon nitride (g-C_3_N_4_), a metal-free semiconductor [60]. In the presence of the g-C_3_N_4_ photoinitiator, straight chain polyacrylate and polyacrylamide were obtained via PET–RAFT polymerization, without previous deoxygenation. The polymers exhibited narrow polymer dispersities and high end-group fidelity. Polymer growth was noted to occur only under light exposure; it was arrested in the absence of irradiation. 

Moreover, Jiang et al. investigated RAFT polymerization of methyl methacrylate (MMA) using another type of metal-free photoinitiator (i.e., carbon dots (CDs)) [61]. Several heteroatoms, including chalcogens and pnictogen-doped carbon dots were evaluated; the P- and S-doped CDs were found to be effective photocatalysts under visible light for RAFT polymerization after a photoinduced-electron transfer (PET) process that involved an oxidative quenching mechanism. The CDs showed strong absorbance of ultraviolet light due to the n–π*(C=O) and the π–π*(C=C) transitions in the CD particles. Temporal control over polymerization was noted; in addition, polymers with chain-end fidelity and narrow dispersity (Đ ≈ 1.04) were demonstrated using this approach [61].

Hang et al. studied the role of graphitic carbon-based nanoporous composites in developing UV photocurable coatings [62]. They fabricated graphene carbon nitride/metal organic framework (g-C_3_N_4_/MOF) composites with nanoscale porosity via a solvothermal approach. The average particle size of the g-C_3_N_4_/MOF-5 composite structures was observed to be nearly 10–15 μm; the pores in these structures were 10–100 nm in diameter. UV–Vis diffuse reflectance spectroscopy recorded a substantially lower optical band gap for the as-synthesized composite, of 2.69 eV, which was significantly lower than the corresponding value for the MOF-5 material (3.86 eV) [62]. The composite was found to possess strong photocatalytic activity, which was evaluated in terms of the variation in curing time of a UV light-cured coating; nanoporous g-C_3_N_4_/MOF-5 composites were shown to shorten the curing time to 13 min from 20 min [62].

### 2.3. Graphene-Based QPIs

Graphene sheets are known to exhibit extraordinary electronic transport properties, thermal conductivity, mechanical stiffness, and fracture strength [19]. Graphene was physically dispersed into polymer precursors; polymerization of this material was performed under UV illumination. Rapid transformation of a liquid monomer into a solid film with tailored mechanical properties and physical–chemical properties was demonstrated [22]. Recent studies on graphene derivatives have demonstrated the ability of graphene oxide (GO) to initiate radical polymerization of acrylic monomers on thermal or photochemical reduction [18,19,20,21,22]. The findings have opened new routes to produce polymer-GO hybrid composites with pH-responsive behavior, high electrical conductivity, improved thermal stability, and exceptional absorbency. Polymers such as polyvinylpyrrolidone or polyvinyl acetate grafted with graphene oxide exhibit good solubility in organic solvents and may be utilized for this approach [18,19,20,21,22,23].

Andryushina et al. investigated the photopolymerization using UV-visible light of acrylamide in aqueous solutions that included colloidal graphene oxide [20]. Graphene oxide was first observed as a water-soluble photoinitiator for polymerizing acrylamide. It was observed that the efficiency of polymerization was associated with the activity of photoexcited oxygen functionalities of GO; the higher the density of these groups, the greater the growth of the polymer. [20]

**Figure 4:**
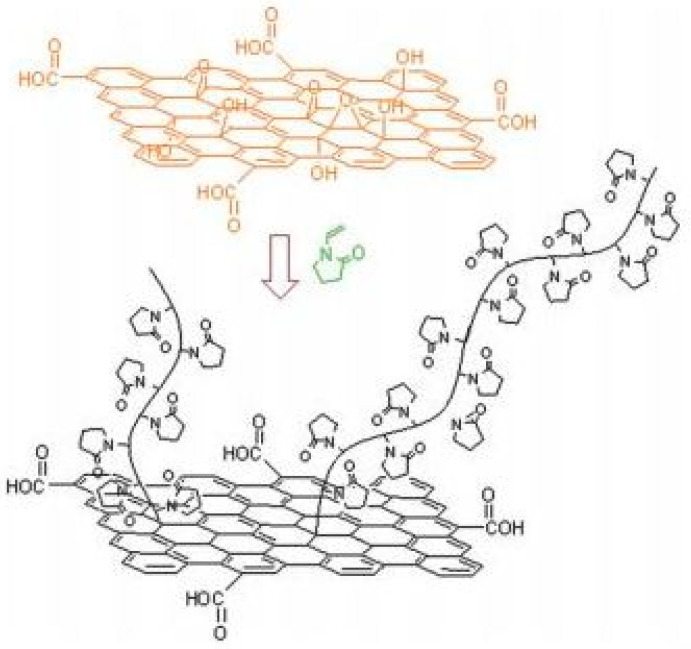
GO surface-initialized polymerization of N-vinylpyrrolidone [19].

Feng et al. demonstrated the fabrication of superhydrophobic surfaces from a formulation containing the I-907 free-radical photoinitiator, thiol-coupled graphene nanosheets, ethoxylated bisphenol A diacrylate, and 2-(perfluorooctyl) ethyl acrylate, which were prepared via a photopolymerization process [21]. Light-induced cross-linking between the reactive graphene nanosheets and the reactive monomers led to the formation of a robust self-wrinkling surface morphology, due to a UV curing process-generated inner tension within the composite (Figure 4). The presence of residual fluorine groups enabled strong cohesive forces, leading to the growth of surfaces with oleophobicity, as well as superhydrophobicity. Using this approach, a coating with a nonstick appearance was obtained. The superhydrophobic character of the material was maintained at extreme pH conditions (1–12); the material was able to withstand a prolonged UV-irradiation time of 120 h. This approach may enable the large-scale fabrication of surfaces with superhydrophobic properties [21].

Sangermano et al. investigated the distribution of water-dispersible graphene oxide sheets in a poly(ethylene glycol) diacrylate (PEGDA) polymeric resin, which were made by a photopolymerization process via UV curing [22]. Under UV illumination, the GO nanosheets became soluble in the aqueous solution of monomers, to form a precursor mixture having low viscosity and high consistency. The study indicated that the graphene sheets were well spread in the matrix of the graphene/poly(ethylene glycol) diacrylate resin composite system. Using this approach, a uniformly transparent composite with substantially similar thermal properties, as compared with the bare resin, was obtained. The composite showed electrical conductivity, even at a very low loading ratio of 0.02 wt% of graphene oxide. The photopolymerization coating strategy may be used to produce products such as antistatic coatings and electromagnetic shielding [22].

A similar approach was adopted by Wang and coworkers, in which graphene nanosheets underwent surface functionalization via simple covalent linking of graphene with 3-methacryloxypropyl trimethoxysilane (MPTES), to give functionalized graphene nanosheets (f-GNS) [23]. The f-GNS and acrylate monomer solution was exposed to UV irradiation; it underwent a photocuring process to produce functionalized graphene/polyurethane acrylate (f-GNS/PUA) nanocomposites. Using a f-GNS content of 1 wt%, the polymer nanocomposite was found to have increased the onset of thermal degradation by 16 °C. The storage modulus values and glass transition temperature values of the nanocomposites were improved by the inclusion of f-GNS in PUA. This phenomenon, which is attributed to the covalent functionalization of graphene, may improve f-GNSs-PUA interactions and the dispersion of f-GNSs in the polymer matrix [23].

### 2.4. UCNPs and Hybrid QPIs

**Figure 5:**
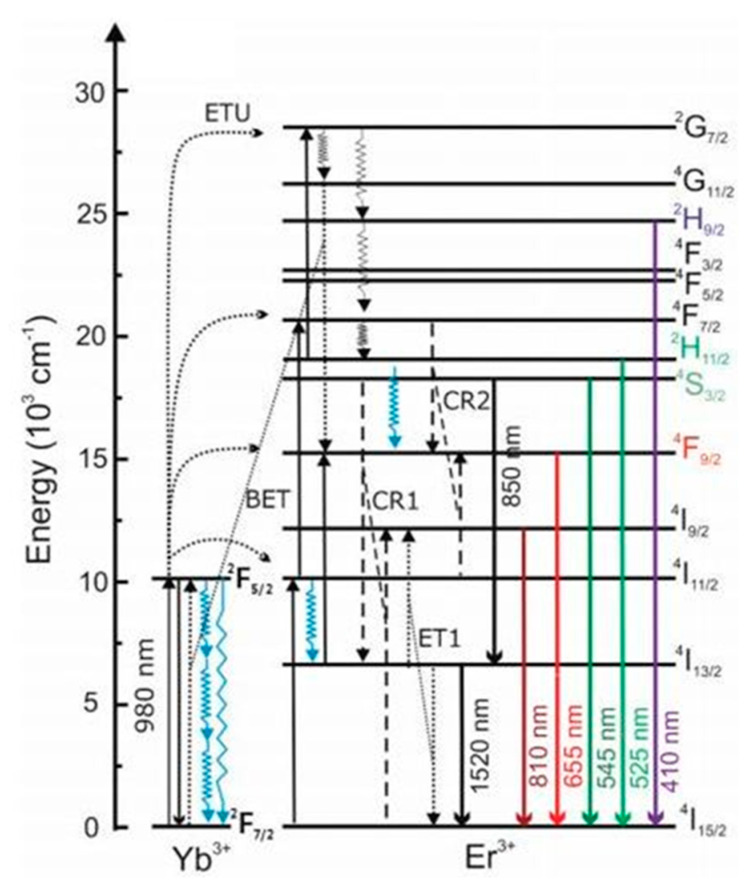
Energy scheme of Yb^3+^, Er^3+^ co-doped NaYF4; ET: energy transfer, ETU: energy transfer upconversion, CR: cross relaxation, and BET: back energy transfer. The Yb^3+^ ground state absorption (GSA) processes do not generally occur at the same Yb^3+^ ion or at neighboring Yb^3+^ ions, yet the absorbed energy migrates from the absorption site to Yb^3+^ ions adjacent to Er^3+^ upconverting centers. Thus, the Yb^3+^energy level diagram represents Yb^3+^ in the NaYF_4_ lattice, whereas the Er^3+^energy level diagram represents a single Er^3+^ ion. Right, top panels: P-dependence of the photon upconversion luminescence of DSPE-stabilized UCNPs in water and D_2_O. Exemplarily shown are spectrally corrected emission spectra of DSPE-capped UCNPs in water (black lines) and D_2_O (red lines) at low P of ca. 16 W cm^−2^ [63].

Upconverting nanoparticles (UCNPs) are luminescent materials that are capable of emitting higher energy, lower wavelength photons on absorption of lower energy, higher wavelength incident light [24,25,26,27,28,29,30,31,32,33,34]. This photophysical phenomenon, which is referred to as photon upconversion, is based on an anti-Stokes process and involves sequential absorption of two or more low-energy photons, which enables the population of real, intermediate excited electronic states, followed by emission of a single high-energy photon. The commonly studied UCNPs are based on a NaYF_4_ crystalline host matrix, which includes activator (e.g., Er^3+^ and Tm^3+^) lanthanide ions, as well as an upconversion sensitizer (e.g., Yb^3+^). The UCNPs showed anti-Stokes shifted visible, as well as ultraviolet, emission with a minimal autofluorescence background after excitation of the UCNPs by near-infrared (NIR) continuous wave laser light [24,25,26,27,28,29,30]. Recently, a number of reports have suggested these UCNPs possess low quantum yields, and enhancement in up conversion luminescence can be effectively achieved by doping with plasmonic semiconductor nanocrystals (Figure 5). Liu et al. reviewed several strategies to increase up conversion luminescence efficiency, such as energy transfer modulation, surface passivation, host lattice manipulation, photonic crystal engineering, broadband sensitization, and surface plasmonic coupling. Rationally designed nanohybrids of UCNPs with enhanced photocatalytic activity behave as effective panchromatic radical photoinitiators [31,32,33,34].

Chen et al. evaluated the use of thulium (Tm)- and ytterbium (Yb)-based UCNPs (e.g., NaYF_4_:TmYb@NaYF_4_) in combination with a known organic photoinitiator, bis(4-methoxybenzoyl) diethyl german [24]. These UCNPs sensitized the photolytic cleavage of the organic blue/UV photoinitiator and initiated radical photopolymerization after exposure to light from a λ = 974 nm near-infrared (NIR) laser. The UCNP/photoinitiator system was compared with a metal-free photo-atom transfer radical polymerization (ATRP) system, which consisted of all organic photoinitiators *iso*-propyl thioxanthone (ITX), *N*,*N*,*N′*,*N″*,*N″*-pentamethyldiethylenetriamine (PMDETA) and α-bromo(*iso*-butyl) ethylester (α-BrBuEt). The former system showed significantly larger polydispersity in contrast to the latter. Chain extension analysis indicated that polymerization may be effectively managed by the controlled chain termination step. The process resulted in crosslinking of 1, 6-hexanediol diacrylate (HDDA) using UCNPs and excitation from a NIR laser. The interference from the UV filter materials, such as TiO_2_, was shown to not inhibit crosslinking during the curing process [24].

Li et al. investigated the light-induced polymerization of thick pigmented systems, which often exhibit a light screening effect [25]. They demonstrated a facile approach based on UCNPs-led photopolymerization of a difunctional bisphenol A epoxy acrylate oligomer called CN104A80 that allowed for an adequate curing depth; the photoinitiator system was sensitized to initiate radical polymerization when the UCNPs converted NIR light to higher energy UV and visible light on illumination with a 980 nm laser. The optimized formulation contained 0.7 wt% of the photoinitiator Irgacure 784, 0.5 wt% red pigment, and 0.9 wt% UCNPs for polymerization of the CN104A80 resin. The double bond conversion was noted to be nearly 70%, and the photopolymerization depth in the processed material was shown to be around 25.5 mm. The peak temperature during UCNPs-assisted photopolymerization, 120.4 °C, was comparable with the temperature used for frontal photopolymerization of composite polymeric materials. Materials processed using UCNP-led photocuring showed higher hardness and modulus values than the materials that were processing using blue LED light [25]. 

Rocheva et al. studied the photopolymerization of a mixture containing poly(methyl methacrylate) (PMMA) and the oligocarbonate methacrylate (OCM-2) for 3D rapid prototyping in the presence of photocurable composition (PCC) containing UCNPs and using a semiconductor laser [26]. UCNPs were designed so as to yield a large conversion efficiency in the UV range. Core/shell UCNPs, e.g., NaYF_4_:Yb^3+^, Tm^3+^/NaYF_4_, with PCC and distinct ultraviolet-emitting light exhibited an ultraviolet conversion efficiency of η_UC^((UV)) = 2%. In addition, light-sensitive resins containing photoinitiators were processed using NIR excitation below 10 W cm^−2^, which induced the formation of radicals and photopolymerization in situ [26].

In another study, Oprych et al. studied the photopolymerization of urethane dimethacrylate with core/shell type UCNPs that emitted photons of blue and UV light on absorption of laser light at 980 nm [30]. The core consisted of Tm and Yb doped NaYF_4_:Yb^3+^/Tm^3+^, while the host lattice of the shell was kept deliberately un-doped. Three radical photoinitiators (camphorquinone, thioxanthone, and vocerin) and one coinitiator, sulfonium salt were evaluated in this study. The UCNP systems in combination with a 980 nm laser with a line-shaped focus were used for photoexcitation of the material. This material-laser combination was used to be suitable for use in yellow safe-light conditions [30]. The study also described the development of UCNPs with a chemically bound thioxanthone; this approach places the photoinitiator in close proximity to the site where the excitation light is generated. Jee et al. also studied the photopolymerization of monomers of the highly branched epoxy photoresist called SU-8 in the presence of NaYbF_4_:Tm^3+^ core-based UCNPs, which exhibited upconversion photoluminesence in NIR, visible, and UV light [32]. Polymerization of nanocomposite structure by 980 nm irradiation from a laser diode was demonstrated using this approach.

Zhang et al. reported on the fabrication of an efficient radical photointiator with the ability to initiate photopolymerization from the shorter wavelength region to the longer wavelength region [31]. A triple core-shelled structure (Ag@SiO_2_@UC@BFO-Au) containing a AgNP core was created using UCNPs, and silver (Ag) and gold (Au) plasmonic nanoparticles (NPs), as well as BiFeO_3_ (BFO) semiconductor nanocrystals. This UCNP material could initiate polymerization after exposure to visible or NIR light at room temperature; moreover, this photoinitiator can undergo recycling for subsequent reuse [31].

**Figure 6:**
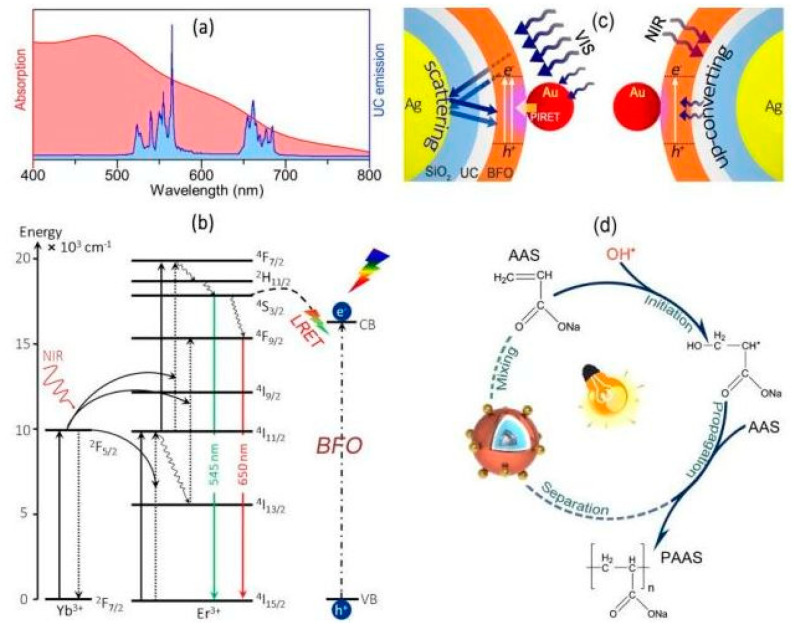
(**a**) Schematic illustration of spectral overlap of the absorption (red) of the BFO shell and emission (blue) of the upconverting core. (**b**) Energy level diagrams of Yb^3+^ and Er^3+^ ions, as well as the upconverting luminescence process upon NIR excitation; the energy transfer from upconverting core to BFO shell. (**c**) SPR enhanced the photoactivity of Ag@SiO_2_@UC@BFO-Au under VIS and NIR excitation. (**d**) Schematic process of the AAS photo-polymerization using Ag@SiO_2_@UC@BFO-Au as a photoinitiator [31].

The activity of this Ag@SiO_2_@UC@BFO–Au nanohybrid photoinitiator was associated with the synergistic effect of the combined optical properties of upconverting and the surface plasmon resonance (SPR) phenomenon in the core@shell configuration; the Ag NP core increased the resonant visible light photon scattering in a “mirror” role. In addition, it improved the upconverting efficiency of NIR photons to visible photons in a “resonator” role. The Au NPs within the BFO shell enabled an efficient energy transfer (Figure 6) of absorbed visible photon energy to BFO from Au through plasmon-induced resonance energy transfer (PIRET) [31]. The photopolymerization of acrylic acid sodium (AAS) using the Ag@SiO_2_@UC@BFO–Au nanocomposite was demonstrated at room temperature; photopolymerization was noted to occur under near infrared and visible light irradiation [31]. The photoinitiator showed excellent stability, as well as the capability for being separated and reused [31].

Ding et al. studied another photopolymerization technique, called reversible deactivation radical polymerization (RDRP), which involved the combined role of dithiocarbonyl compounds, which served as the initiator-mediator, and UCNPs, which served as internal lamps on illumination with a NIR light source [33]. Several monomers with ester functionalities, such as vinyl acetate, butyl acrylate, and methyl methacrylate were polymerized through this procedure. Matrix-assisted laser desorption/ionization time-of-flight mass spectrometry studies and nuclear magnetic resonance studies showed that the polymers exhibited excellent end-group fidelity. NIR light was noted to be an attractive light source for photo-induced RDRP, due to its low energy and strong penetration capability [33].

### 2.5. Polymer–Hybrid QPIs

Generally, organic compounds are used as Type I and II photoinitiators in UV–Vis curing technology for the fabrication of functional polymeric materials, including inks, coatings, and adhesives. Type I photoinitiators are known to undergo α-cleavage and decompose into two radical species under UV–Vis light irradiation [64] and type II photoinitiators form radicals via a multi-step reaction process in the presence of co-initiators. Amines are commonly used as a co-initiator; an electron is transferred to the triplet state in the type II initiator, resulting in proton release to form radicals. Commercially available type II initiators with broad wavelength absorption over UV-Vis wavelengths include thioxanthone (TX) and its derivatives, benzophenone (BP) and camphorquinone (CQ). However, these compounds are relatively small in size and can enter the polymer matrix while cross linking. Thus, the small molecular type I photoinitiators are often attached to polymeric nanoparticles to produce efficient photoinitiator systems [64].

**Figure 7:**
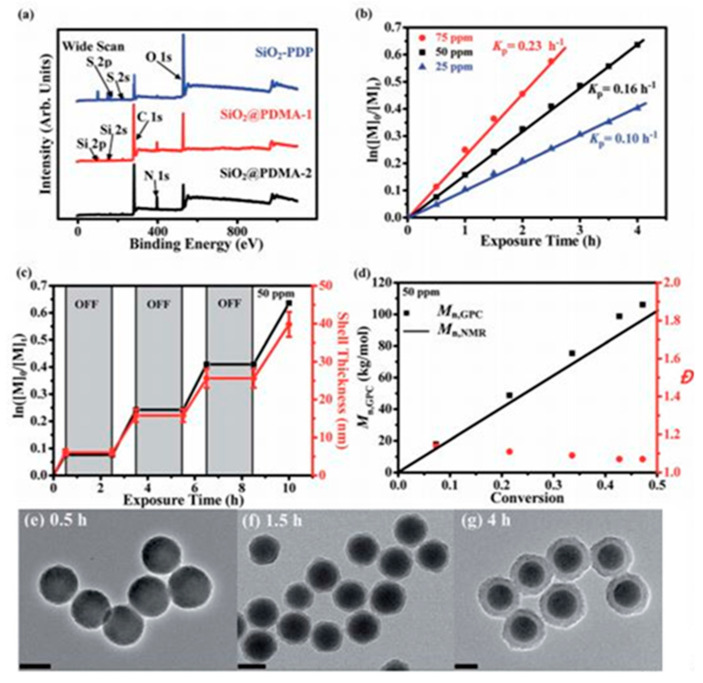
Surface-initiated PET-RAFT polymerization of DMA monomers from SiO_2_-PDP nanoparticles in acetonitrile with prior deoxygenation at 25 °C under blue LED light irradiation (4.8 W, λ max ¼ 465 nm, 1.0 mW cm^2^). (**a**) XPS wide-scan spectra of SiO_2_-PDP, SiO_2_@PDMA-1, and SiO_2_@PDMA^-2^ nanoparticles; (**b**) plot of ln[M]0/[M]t versus exposure time t at three different Ru(bpy)_3_Cl_2_ concentrations with reference to monomer concentration; (**c**) temporal control over polymerization upon on/off switching of light; and (**d**) evolution of Mn,NMR, Mn,GPC, and Đ versus monomer conversion. TEM images of (**e**) SiO_2_@PDMA-1, (**f**) SiO_2_@PDMA-2, and (**g**) SiO_2_@PDMA-3 nanoparticles. All scale bars are 100 nm [66].

Du et al. demonstrated an strategy involving the combination of a type I photoinitiator and the nanoparticles of a functionalized block copolymer through reversible addition–fragmentation chain transfer (RAFT)-mediated polymerization-induced self-assembly (PISA) in an aqueous medium; the choice of the block copolymer nanoparticle affects the properties of the hydrogel that is prepared (Figure 7). Using this approach, hydrogels with embedded nanoparticles may be prepared using block copolymer nanoparticle-based heterogeneous photoinitiators [64].

Lu et al. investigated control/living radical polymerization (CLRP) using long-wavelength light [65]. They successfully demonstrated the polymerization of methyl methacrylate (MMA) for the synthesis of polyMMA (PMMA) with a narrow molecular weight distribution, using photoinduced atom transfer radical polymerization (ATRP). One-dimensional nanopoly(dip henyl butadiyne) (nanoPDPB) was used as a photocatalyst, which produced radicals via activation of a dormant alkyl bromide initiator [65]. Light-regulated initiation, as well as termination of polymerization, were demonstrated using this approach. The radicals created via the redox reaction of EBP and nanoPDPB enabled the polymerization of MMA [65]. 

**Figure 8:**
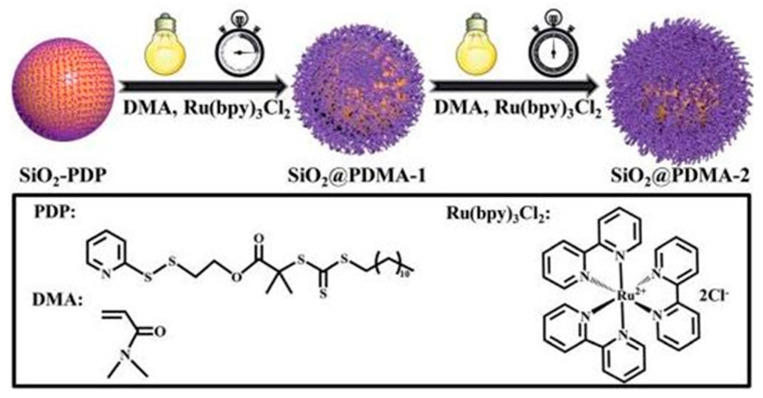
Surface-initiated PET–RAFT polymerization of DMA monomers from SiO_2_-PDP nanoparticles in acetonitrile, with prior deoxygenation at 25 °C under blue LED light irradiation [66].

Li et al. described polymer brush growth from the surface of silica nanomaterials via the PET–RAFT approach [66]. Through spatiotemporal control over light regulation, silica nanocomposites that were coated by polymer brushes with narrow molecular weight dispersities and high grafting densities were prepared; for example, surface-initiated polymerization of DMA monomers from SiO_2_-PDP nanoparticles under blue LED light irradiation using the PET–RAFT approach was demonstrated (Figure 8) [66].

Co-initiators in the system were shown to accelerate the process of radical generation and increase the efficiency of the photopolymerization process (i.e., the polymer chains formed per excitation); triethylamin, which was associated with quantum efficiencies of initiation of greater than 5%, was noted to be the most effective [8]. Molecularly passivated QDs were noted to exhibit large TPA cross-sections and modest quantum efficiencies, making them appropriate two-photon photosensitizers.

## 3. Applications of Photopolymers Based on QPIs

Nazim et al. investigated the role of a photopolymerized polymeric thin film in constructing a copper indium gallium selenide solar cell with high efficiency [67]. The group utilized nitrogen-doped graphene quantum dots with transparency, highly emission, and UV-curable properties to fabricate highly dispersed Norland Optical Adhesive (NOA) nanocomposite-based polymeric thin films referred to as poly-QD films [67,68,69,70,71,72,73,74,75,76,77,78,79]. These flexible films served as luminescent downconversion (LDC) layers, which enhanced the function of copper indium gallium selenide solar cells. *N*-graphene quantum dots (GQDs) were embedded within UV-curable NOA matrices; a “click” reaction of thiol–ene components was performed with UV light. The most functional cast poly-QD film showed an efficiency of ∼9.70% in the high-energy solar spectrum (compared with 8.77% for bare copper indium gallium selenide (CIGS) solar cells) [67].

**Figure 9:**
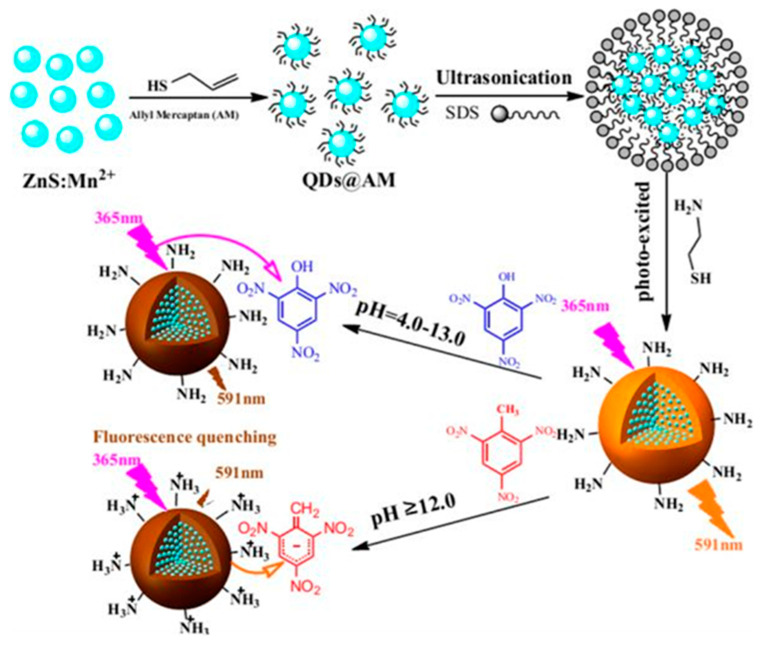
The fabrication of hydrophilic fluorescence nanocomposites (NCs) and the selective detection of TNT or TNP under optimal pH [68].

Bai et al. utilized hydrophilic nanocomposites fabricated from photopolymerization for the highly sensitive and selective detection of polluting nitroaromatics [68]. Hydrophilic amine-functionalized nanocomposites were processed using a light-induced in situ polymerization approach; hydrophobic fluorescence quantum dots that contained ZnS:Mn^2+^@allyl mercaptan (QDs@AM) building blocks were used in this study (Figure 9). The average particle diameter of these as-synthesized hydrophilic nanocomposites was found to be ∼50 nm, which could be further tailored by optimizing the concentrations of the monomers. The linear ranges for detection of 2,4,6-trinitrotoluene (TNT) and 2,4,6-trinitrophenol (TNP) were 0.01–0.5 μg/mL and 0.05–8.0 μg/mL, respectively; little interference by nitrobenzene (NB) and 2,4-dinitrotoluene (DNT) in detection of the analytes was noted [68].

**Figure 10:**
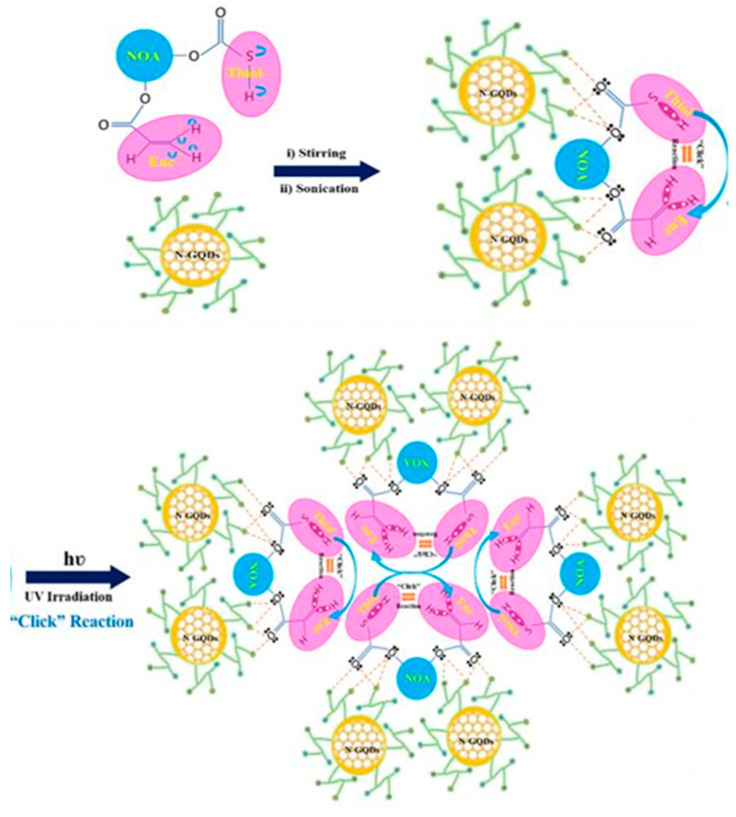
Model of the thiol−ene “click” reaction pathway of NOA polymers with N-GQDs [69].

Nazim et al. investigated the role of photopolymerization to generate a hydrophobic nanocomposite-based polymeric thin film hybrid [69]. The polymer–quantum dot nanocomposite films were synthesized via a simple one-pot, two-step procedure, in which nitrogen-doped graphene quantum dots (N-GQDs) were dispersed in a homogeneous manner within a UV-curable polymer host matrix via a thiol–ene “click” reaction (Figure 10). The NOA–NGQD nanocomposite films showed hydrophobic behavior, with water contact angle values greater than 69. Several heteroatom functionalities were introduced on the N-GQD surface (e.g., amine, carbonyl, and hydroxyl groups), which served as sites for electrostatic interactions between N-GQDs and NOA that led to the formation of flexible NOA–NGQDs nanocomposites [69]. The NOA–NGQD nanocomposites demonstrated high transparency (>90%) and a low band gap [72]. Schmitt et al. investigated the role of nanoscale ZnO with non-photo-reactive levulinic acid for initiating the polymerization of an acrylic ester mixture; the total amount of levulinic acid and the acidity of the reaction solution were shown to affect the photopolymerization process [6].

Hampton et al. demonstrated the pattern replication in nonwetting templates (PRINT) process to obtain pattern replication with 2.7 nm diameter CdSe quantum dots (QDs) and a pyridine surface ligand [70]. They prepared two-dimensional CdSe arrays on indium-doped tin oxide (ITO) electrodes using this process [70]. The PRINT technique utilized an elastomeric mold based on perfluoropolyether (PFPE), which enabled patterning of CdSe QD solutions without mold alteration. Nanometer-scale diffraction gratings were successfully replicated with CdSe QDs using this approach [70].

**Figure 11:**
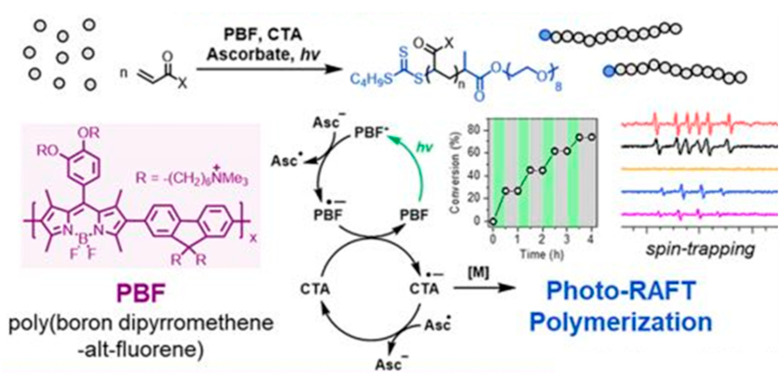
Photo-controlled RAFT polymerization of acrylic monomers, using the conjugated polymer PBF as the photocatalyst [80].

Lu et al. employed poly(boron dipyrromethene-alt-fluorene) (PBF), a conjugated polymer with greater photostability than eosin Y, as a photocatalyst for photo-RAFT polymerization of acrylic monomers (Figure 11) [80]. The reaction progress was controlled by modulating the irradiation conditions. The optical spectroscopy and electron spin resonance results of this study indicated that the reductive quenching of PBF by ascorbate was associated with the reduction of a chain transfer agent [80].

Allan et al. investigated the light-selectivity of the photopolymerized polymer films containing poly(ethylene-vinyl acetate) (PEVA) [71]. These films are of significant interest as solar materials, such as greenhouse plastic films and PV encapsulation materials; for example, QDs that absorb UV light and transmit light of higher wavelengths may be used to enhance PV efficiency or improve greenhouse functionality [71]. A simple melt-mixing in a twin-screw extruder was used to load QD nanocrystals into PEVA [71]. Both bare CdS and core–shell CdS–ZnS QDs were prepared using colloidal chemistry using a single-molecule precursor; functionalization with a silane coupling agent such as (3-mercaptopropyl) trimethoxysilane was used to improve their compatibility with PEVA. The silane ligand was shown to enhance the dispersibility of QDs in the PEVA material [71,72,73]. 

Sajjad et al. demonstrated a dual catalytic system containing conjugated microporous polymers (CMP) of phenothiazine (PTZ-CMP) as a heterogeneous photocatalyst and a Cu catalyst; ATRP was performed on exposure to red or green light [79]. They showed that PTZ may be modified to generate heterogeneous photocatalysts for ATRP of methacrylate and acrylate monomers; this process was mediated by the Cu complexes [79]. The CMPs exhibited photocatalytic activity on green light irradiation. Crosslinking the PTZ units via aromatic linkages were associated with conjugation within the network, as well as visible light activity [79]. The ATRP activator species were prepared by photoinduced redox reactions; the polymerization was mediated by the level of the ATRP catalyst. The heterogeneous nature of the photocatalyst enabled separation and reuse for further polymerization, without a decrease in photocatalytic efficiency [79].

Wang et al. studied the use of silicon quantum dots (SiQDs) as photocatalysts for PET–RAFT polymerization under visible light irradiation [81]. They investigated the effect of the polarity of the solvent, reducing agent, wavelength of irradiation light, and various monomers to understand the efficiency of SiQDs as a photocatalyst. Functionalization of the SiQDs with an amino group, which served as a bridge between substrates and terminal carboxyl group chain transfer agents (CTAs), facilitated surface-initiated PET–RAFT polymerization [81]. 

Yifan et al. created well-dispersed polymer-QDs nanocomposites via RAFT polymerization, mediated by CdSe QDs as the photocatalyst [82]. CdSe QDs are efficient photoredox catalysts for light mediated ATRP and free radical polymerization; moreover, the CdSe QDs excited-state exhibits a strong reducing potential (−1.59 V vs. saturated calomel electrode) to mediate PET–RAFT polymerization using a electron transfer mechanism similar to ATRP; the RAFT agents were noted to decorate the surface of CdSe QD [82]. Initiation and propagation of the polymer chain occurred from the CdSe QD surface; the CdSe QDs served both as the inorganic nanocomposite and the photocatalyst [82].

## 4. Conclusions

The research on nanocrystals as photocatalysts in general, and specifically as radical initiators, has led to their utilization as quantum photoinitiators for reversible deactivation radical polymerization, free radical polymerization, and photoinduced atom transfer radical polymerization. The potential advantages of quantum PIs, including low migration, blue light excitation, and low energy activation barrier properties, support more efficient radical generation, rapid propagation of the chain reaction, and cross-linking of monomers with a low rate of recombination of charge carriers. The impressive plasmonic, panchromatic, and upconverting properties of quantum PIs have been incorporated into a variety of nanocrystals. Due to the unique features of quantum-sized initiators, these materials will have an increasing role in large-scale polymerization processes and commercial polymerization processes over the coming years.

## Data Availability

All the data will be available to the readers.
